# Prognostic factors and treatment comparison in small cell neuroendocrine carcinoma of the uterine cervix based on population analyses

**DOI:** 10.1002/cam4.3326

**Published:** 2020-07-24

**Authors:** Li‐mei Lin, Qin Lin, Jun Liu, Ke‐xin Chu, Yun‐Xia Huang, Zong‐Kai Zhang, Tao Li, Ya‐Qing Dai, Jin‐Luan Li

**Affiliations:** ^1^ Department of Radiation Oncology Xiamen Cancer Center The First Affiliated Hospital School of Medicine Xiamen University Teaching Hospital of Fujian Medical University Xiamen China

**Keywords:** gynecological oncology, prognosis, radiotherapy, SEER

## Abstract

**Objective:**

We aimed to assess the impact of the treatment modality on the outcome of small cell neuroendocrine cervical carcinoma (SCNEC) using the Surveillance Epidemiology and End Results (SEER) database.

**Methods:**

Patients from the SEER program between 1981 and 2014 were identified. Significant factors for cancer‐specific survival (CSS) and overall survival (OS) were analyzed using the Kaplan‐Meier survival and Cox regression methods.

**Results:**

A total of 503 SCNEC patients were identified. The 5‐year CSS and OS were 36.6% and 30.6%, respectively. The International Federation of Gynecology and Obstetrics (FIGO) stage I to IV distributions was 189 (37.6%), 108 (21.5%), 95 (18.9%), and 111 patients (22.0%), respectively. Within the patients with known treatment strategies, 177 (45.9%) were treated with radical surgery and 209 (54.1%) underwent primary radiotherapy. Local treatment strategies were independent prognostic factor for CSS and OS. The 5‐year CSS for radical surgery and primary radiotherapy was 50.0% and 27.9%, respectively (*P* < .001). The 5‐year OS for those who received radical surgery and primary radiotherapy was 57.8%, and 29.6%, respectively (*P* < .001). In FIGO stage I SCNEC, patients treated with radical surgery had superior CSS (*P* = .001) and OS (*P* = .003) than those with primary radiotherapy. However, in FIGO stage II and III SCNEC, there were no differences in CSS and OS with respect to different local treatment strategies. Our results also found that the addition of brachytherapy impacted OS in the FIGO stage III SENCE (*P* = .002). The 5‐year CSS and OS of patients with FIGO IV were only 11.7% and 7.1%, respectively.

**Conclusions:**

SCNEC is a rare disease with aggressive clinical behavior. The findings indicate that radical surgery should be suggested for early‐stage SCNEC and combining radiation therapy with brachytherapy should be suitable for patients with advanced stage.

## Graphical abstract

SCNEC is a rare disease with aggressive clinical behavior. The findings indicates that radical surgery should be suggested for early‐stage SCNEC and combining radiation therapy with brachytherapy should be suitable for patients with advanced stage.

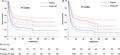

## 1 INTRODUCTION

Nearly 2 to 5% of all cervical carcinomas are small cell neuroendocrine uterine carcinomas (SCNEC).^1^, ^2^, ^3^ Compared with common histological types, SCNEC seems to be highly aggressive and has a worse prognosis even in its early stages.^4^, ^5^, ^6^, ^7^, ^8^, ^9^

Because of the rarity of the disease, most of the previous studies were clinical case‐reports or limited series and from single institutions. To date, there are no published guidelines for the standard local therapy for SCNEC. Prognostic factors including age, tumor size, tumor stage, metastases of the lymph node, and margin status were examined with various results. The 5‐year survival of early‐stage tumors was 30%‐46%, and only 0%‐15% in advanced‐stage carcinoma.^10^ The therapeutic approach for SCNEC still remains a challenge.

Given the aggressive clinical behavior of the disease, to improve treatment outcome a potential treatment strategy different from that of common histological types is required. Therefore, the aim of our research was to estimate the effect of treatment modality on survival in SCNCE using the Surveillance Epidemiology and End Results (SEER) database.

## 2 PATIENTS AND METHODS

### 2.1 Data source

Our research data were extracted from the SEER database maintained by the National Cancer Institute. The SEER database is composed of 18 population‐based cancer registries and covers nearly 27.8% of the US population (based on the 2010 Census). We accessed data files with the SEER ID 13027‐Nov2018 and identified patients with a primary diagnosis of SCENC between 1981 and 2014. The primary site was used for the pathological diagnosis of the illness according to the third edition of the International Oncology Classification (ICD‐O‐3). We identified local treatment strategies using codes for surgery and radiotherapy (RT). Radical hysterectomy, modified radical hysterectomy, complete hysterectomy, and pelvic exenteration were included in curative surgery. External beam radiation therapy (EBRT), with or without brachytherapy before curative surgery, was defined as local radiation treatment.

### 2.2 Clinicopathological factors

The clinicopathological and demographic variables were gathered as follows: age of diagnosis, race, tumor grade, International Federation of Gynecology and Obstetrics (FIGO) stage, tumor size, state of lymph node, and treatment strategies including radical surgery and primary RT. Duration of follow‐up and vital status, including the cause of death was also included. The primary endpoints were CCS and OS.

### 2.3 Statistical analysis

We used univariate and multivariate Cox proportional hazards models to estimate prognostic factors for CSS and OS. Factors that were significantly related to CSS and OS in univariate analyses were included in the multivariate analysis. Calculation of CSS and OS were evaluated using the Kaplan‐Meier survival and Cox regression proportional hazard methods. All calculations were carried out with the SEER‐Stat software (SEER*Stat 8.3.5). Two‐sided *P*‐values were calculated and a cutoff of *P* < .05 was statistically significant.

## 3 RESULTS

### 3.1 Patient characteristics and treatment

A total of 503 SENCE patients were reported in the SEER database from 1981 to 2014. The demographic, clinicopathological, and treatment characteristics of the population are listed in Table 1. The median follow‐up time was 19 months (range 3‐323 months). The FIGO stage I to IV distributions were 189 (37.6%), 108 (21.5%), 95 (18.9%), and 111 patients (22.0%), respectively. A total of 341 patients with known histologic grade were available, 336 had poorly or undifferentiated grade. Of the 189 patients for whom data on tumor size were available, 53 had tumor size < 4 cm, and 136 cases ≥ 4 cm (Table 1). Among the 386 patients with known local treatment strategies, 177 patients were treated with radical surgery and 209 patients underwent primary RT. Subsequent RT was performed in 113 patients who received radical surgery. Of the patients that underwent primary RT, 129 were treated with beam radiation and 80 received a combination of brachytherapy. A total of 374 patients had received chemotherapy. Of the 172 patients who received lymphadenectomy, there were 88 with nodal metastases.

**TABLE 1:** Patient characteristics

Variables	N (%)
Age (y)	
<50	283 (56.3)
≥50	220 (43.7)
Race	
White	347 (69.0)
Black	80 (15.9)
Other	76 (15.1)
Grade	
Well differentiated	1 (0.2)
Moderately differentiated	4 (0.8)
Poorly/undifferentiated	336 (66.8)
Unknown	162 (32.2)
Stage (FIGO stage)	
I	189 (37.6)
II	108 (21.5)
III	95 (18.9)
IV	111 (22.0)
Local treatment	
Radical surgery	177 (45.9)
With RT	113 (29.3)
Without RT/unknown	64 (16.6)
Primary RT	209 (54.1)
EBRT	129 (33.4)
EBRT + B	80 (20.7)
Chemotherapy	
Yes	374 (74.4)
No	129 (25.6)
Tumor size	
<4	53 (10.5)
≥4	136 (27.1)
Unknown	314 (62.4)
Nodal status	
Negative	84 (16.7)
Positive	88 (17.5)
Unknown	331 (65.8)

Abbreviation: EBRT + B, external beam radiotherapy plus brachytherapy.

The treatment characteristics of the different stages are listed in Table 2. Among the 189 patients with FIGO I stage, 111 (58.7%) were treated with radical surgery, 46 (24.3%) with primary RT, and 137 (72.5%) with chemotherapy. Among the 108 patients with FIGO II stage, 34(31.4%) were treated with radical surgery, 55(50.9%) with primary RT, and 85(78.7%) with chemotherapy. Among the 95patients with FIGO III stage, 15(15.8%) were treated with radical surgery, 60(63.2%) with primary RT, and 78(82.1%) with chemotherapy. Among the 111 patients with FIGO IV stage, 17(15.3%) were treated with radical surgery, 48(43.2%) with primary RT, and 74(66.7%) with chemotherapy.

**TABLE 2:** Treatment characteristics of different FIGO stage

Variables	N	Radical surgery	Primary RT	Chemotherapy
		N (%)	N (%)	N (%)
I	189	111 (58.7)	46 (24.3)	137 (72.5)
II	108	34 (31.4)	55 (50.9)	85 (78.7)
III	95	15 (15.8)	60 (63.2)	78 (82.1)
IV	111	17 (15.3)	48 (43.2)	74 (66.7)

Abbreviations: FIGO, International Federation of Gynecology and Obstetrics; RT, radiotherapy.

### 3.2 Prognostic factors

Univariate analyses showed that age, nodal status, FIGO stage, chemotherapy, and local treatment strategies were significant prognostic factors for CSS and OS (Table 3). Patients who underwent primary RT had worse CSS than those who received radical surgery (hazard ratio [HR]: 2.002, 95% confidence interval [CI]: 1.523–2.634, *P* < .001). Primary RT was also associated with worse OS than radical surgery (HR: 2.156, 95% CI: 1.661–2.783; *P* < .001).

**TABLE 3:** Median, Univariate analysis of cancer‐specific survival and overall survival

Variables	CSS			OS		
	Median survival (months)	HR (95% CI)	*P*	Median survival (months)	HR (95% CI)	*P*
Age						
<50	31	Reference		27	Reference	
≥50	14	1.786 (1.416–2.252)	<.001	12	1.970 (1.588–2.444)	<.001
Race						
White	17	Reference		16	Reference	
Black	24	0.291 (0.032–2.631)	0.272	19	0.757 (0.565–1.014)	.062
Other	29	0.177 (0.024‐1.276)	.086	26	0.614 (0.417‐0.906)	.014
Grade						
Well differentiated	6	Reference		6	Reference	
Moderately differentiated	17	0.291 (0.032–2.631)	.272	17	0.300 (0.033–2.707)	.283
Poorly/undifferentiated	26	0.177 (0.024‐1.276)	.086	23	0.210 (0.029‐1.512)	.121
Stage distribution (FIGO stage)						
I	159	Reference		67	Reference	
II	23	2.077 (1.488–2.900)	<.001	23	1.862 (1.365–2.540)	<.001
III	14	3.045 (2.168‐4.275)	<.001	11	2.902 (2.123‐3.966)	<.001
IV	9	4.828 (3.522‐6.617)	<.001	8	4.644 (3.478‐6.203)	<.001
Local treatment						
Radical surgery	67	Reference		53	Reference	
Primary RT	16	2.002 (1.523–2.634)	<.001	15	2.156 (1.661–2.783)	<.001
Tumor size						
<4 cm	27	Reference		27	Reference	
≥4 cm	25	1.198 (0.741–1.936)	.461	20	1.130 (0.731–1.748)	.583
Chemotherapy						
Yes	25	Reference		21	Reference	
No	17	1.306 (1.008‐1.692)	.044	12	1.353 (1.066‐1.717)	.013
Nodal status						
Negative	137	Reference		128	Reference	
Positive	30	1.855 (1.189‐2.894)	.006	26	1.879 (1.229–2.874)	.004

Note

Unknown data points were removed before performing statistical tests.

Abbreviations: CI, confidence interval; CSS, cancer‐specific survival; FIGO, International Federation of Gynecology and Obstetrics; HR, hazard ratio; OS, overall survival; RT, radiotherapy.

Multivariate analyses showed that advanced stage (HR 1.414, 95%CI 1.136‐1.761, *P* = .002) and treatment by primary RT (HR 1.652, 95%CI 1.258‐2.212, *P* = .042) were significantly related to inferiorCSS; age ≥ 50 years (HR 1.561, 95%CI 1.216‐2.237, *P* = .034), advanced stage (HR 1.356, 95%CI 1.096‐1.678, *P* = .005), and treatment by primary RT (HR 2.030, 95%CI 1.086‐3.795, *P* = .027) were significantly associated with poorer OS (Table 4).

**TABLE 4:** Multivariate analyses of cancer‐specific survival and overall survival

Variables	CSS			OS		
	HR	95% CI	*P*	HR	95% CI	*P*
Age	—	—	—	1.561	1.216‐2.237	.034
FIGO stage	1.414	1.136‐1.761	.002	1.356	1.096‐1.678	.005
Local treatment	1.652	1.258‐2.212	.042	2.030	1.086–3.795	.027

Abbreviations: CI, confidence interval; CSS, cancer‐specific survival; FIGO, International Federation of Gynecology and Obstetrics; HR, hazard ratio; OS, overall survival.

### 3.3 Survival

**FIGURE 1:**
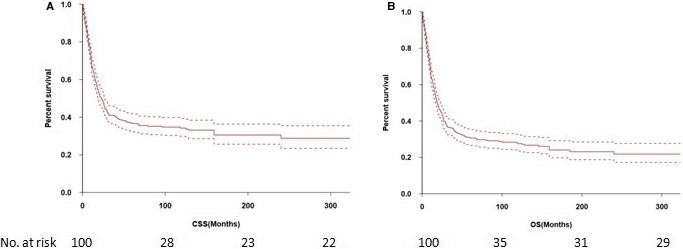
Cancer‐specific survival (A) and overall survival (B) of patients with small‐cell neuroendocrine cervical carcinoma

**FIGURE 2:**
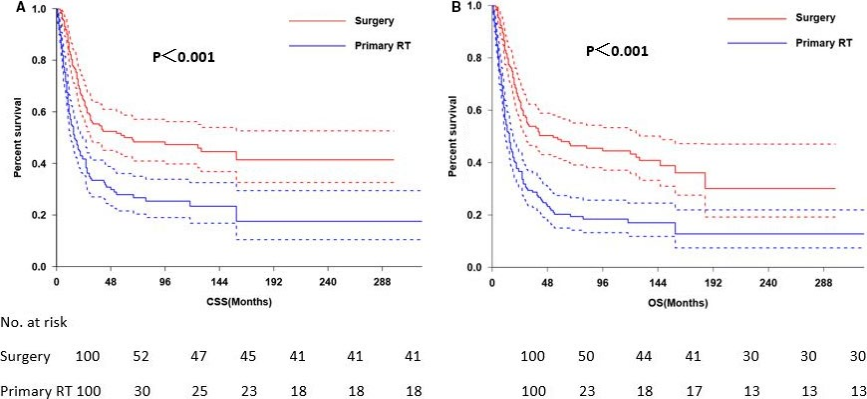
Cancer‐specific survival (A) and overall survival (B) according to local treatment modalities

**FIGURE 3:**
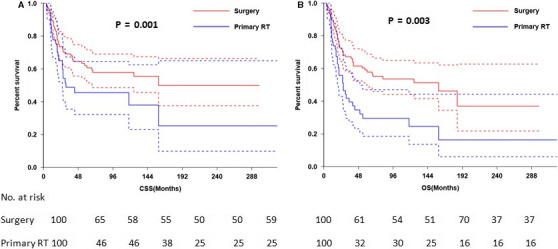
Cancer‐specific survival (A) and overall survival (B) by radical surgery versus primary RT in FIGO stage I patients

**FIGURE 4:**
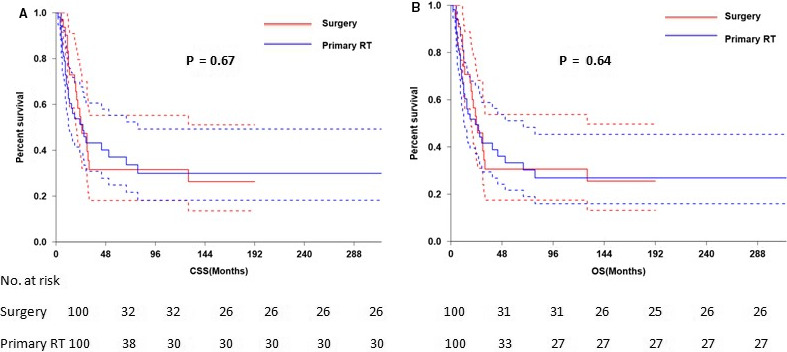
Cancer‐specific survival (A) and overall survival (B) by radical surgery versus primary RT in FIGO stage II patients

**FIGURE 5:**
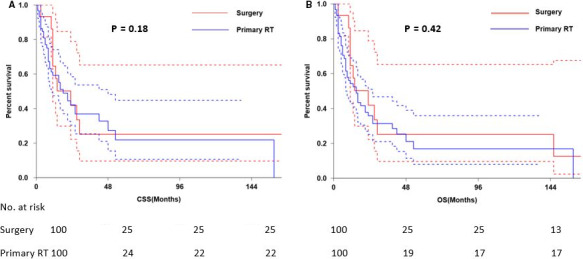
Cancer‐specific survival (A) and overall survival (B) by radical surgery versus primary RT in FIGO stage III patients

The 3‐ and 5‐year CSS were 40.9% and 36.6%, respectively (Figure 1A) and the 3‐ and 5‐year OS were 36.2% and 30.6%, respectively (Figure 1B). The 5‐year CSS in patients treated with radical surgery and primary RT was 50.0%, and 27.9%, respectively (*P* < .001; Figure 2A). The 5‐year OS according to radical surgery and primary RT was 57.8%, and 29.6%, respectively (*P* < .001; Figure 2B). The outcome of the local treatment strategy was different from the FIGO stage. Patients treated with radical surgery had significantly improved CSS (*P* = .001; Figure 3A) and OS (*P* = .003; Figure 3B) compare to those with primary RT in stage I SCNEC. For SCNEC patients with stage II, however, there were no differences in CSS (*P* = .67; Figure 4A) and OS (*P* = .64; Figure 4B), according to different local treatment strategies. There were also no differences in CSS (*P* = .18; Figure 5A) and OS (*P* = .42; Figure 5B) between the patients with stage III according to different local treatment strategies.

**FIGURE 6:**
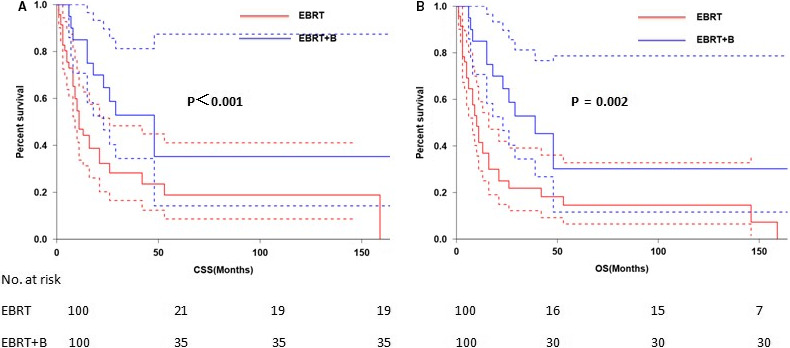
Cancer‐specific survival (A) and overall survival (B) by EBRT vs EBRT plus brachytherapy in FIGO stage III patients

We also investigated the impact of different RT methods on the survival of primary RT‐treated patients and found that RT combined with brachytherapy had an impact on both CSS (*P* < .001; Figure 6A) and OS (*P* = .002; Figure 6B) in stage III SENCE.

## 4 DISCUSSION

This study demonstrated poor CSS and OS for all stages of disease illustrating the aggressive nature of SCNEC. We analyzed the impact of local therapeutic approaches on survival of SCNEC patients from the SEER registry. In our study, the patients with early stages preferred radical surgery and those with advanced stages preferred primary RT. Based on our results, curative surgery would be recommendable as the optimal local therapeutic approach for early‐stage SCNEC whereas combining RT with brachytherapy should be suitable for patients with advanced stage. Currently, there are no prospective studies comparing prognosis in SCNEC patients with different local therapeutic approaches because of the rarity of SCNEC.

There were, however, some reports comparing the survival of SCNEC patients treated with radical surgery versus primary RT which showed different results. In a multicenter study, Chen et al.^11^ observed that in stage I‐II patients, primary RT with aggressive chemotherapy had superior 5‐year OS than surgery (78% vs 40%). In contrast, a systematic review and meta‐analysis concluded that there was no significant difference in the survival outcomes with or without adjuvant RT in SCNEC.^12^ Furthermore, Cohen et al.^13^ previously reported a better OS (38.2% vs 23.8%) in patients with radical hysterectomy. Similarly, another study, by Zhou et al.,^5^ compared survival of SCNEC patients with stage I and II treated with cancer‐directed surgery versus primary RT using the SEER database and showed improved survival after cancer‐directed surgery. However, the results might be affected by the broad definition of cancer‐directed surgery which included biopsy, ablation, and local excision.

In our study, age (≤50 vs >50 years) was found to be an independent factor affecting OS (HR = 1.561, *P* = .034). Several reports have also found a worse survival outcomes with increasing age.^5^, ^7^, ^14^ In a retrospective study of 130 patients with SCNEC, age older than 60 years was proved to be an independent prognostic factors for CSS regardless of stage.^7^ Among the patients with IIB‐IVB stage, those aged younger than 45 years had significantly lower risk of cancer death than those aged 45 to 60 years (HR, 3.4; *P* = .035). Zhou et al.^5^ who analyzed data of 208 patients with stage I‐II SCNEC found that age was an independent prognostic factor for OS (HR = 1.017, *P* = .004). Similarly, Hou et al.^14^ found a significant OS benefit in younger cases (*P* = .005) with high‐grade neuroendocrine carcinoma in a retrospective analysis.

The more advanced stages were found to be correlated with worse survival in several studies.^13^, ^14^, ^15^, ^16^, ^17^, ^18^, ^19^ Zhang et al.^15^ showed that the median survival time (MST) of SCNEC patients with early‐stage (I‐IIA) was longer than those with advanced‐stage (IIB‐IV) (60 vs 30 months, *P* = .016). However, the FIGO stage was not an independent prognostic factor in the multivariate analyses. Another study of the Taiwanese multi‐institutional database concluded that FIGO stage was significantly related to failure‐free survival (FFS) (HR: 2.28, *P* = .001) and CSS (HR: 2.27, *P* < .001) in multivariate analyses.^17^ Similarly, in our study, multivariate analyses showed that FIGO stage was an independent factor affecting CSS and OS (CSS: HR = 1.414, *P* = .002; OS: HR = 1.356, *P* = .005).

Lymph node metastasis probability is reported ranging from 34.9% to 65% in SCENC in several studies,^11^, ^17^, ^18^ which is higher than that reported in common histological types of cervical cancer.^20^ To date, the prognostic value of lymph node status in SCENC is controversial. In this study, lymph node metastasis occurred in 51.2% of SCENC patients, and the status of lymph nodes was proved to be an independent prognostic factor in multivariate analysis. The results were consistent with the research by Wang et al.^17^ in which node metastasis was also selected as an independent variable. Nonetheless, several studies failed to demonstrate the predictive value of lymph node status on survival.^11^, ^13^, ^18^ More studies are warranted to assess the prognostic value of lymph node status in SCENC patients.

Due to the similar biological behavior with small‐cell lung cancer, SCENC was usually treated in a similar fashion as SCLC.^21^, ^22^, ^23^ Several studies have recommended adjuvant chemotherapy or concurrent chemotherapy for SCENC patients according to their results.^11^, ^13^, ^24^, ^25^ Several studies reported that, after 1990, most patients underwent chemotherapy‐dominant comprehensive therapy.^17^, ^24^, ^25^, ^26^ In our study, the chemotherapy was significantly related to favorable CSS and OS in the univariate analyses, however it was not an independent prognostic factor in multivariate analysis. Given that the information about chemotherapy regimens was missing in the SEER database, we were unable to analyze the effect of different chemotherapy regimens on the survival of SCENC patients in this study.

The pathological factors, including parametrial invasion, surgery margin, and lymphovascular invasion are significantly related to survival in cervical cancer. The SEER database did not, however, provide information on the abovementioned pathologic factors. It remains controversial whether the pathological factors in SCENC patients have prognostic value. Wang et al. reported that positive surgical margin was a significant prognostic factor for FFS but not for CSS; parametrial extension and lymphovascular invasion had no prognostic value in multivariate analyses.^17^ On the contrary, Chen failed to identify the surgical margin as a prognostic factor.^11^ Parametrial involvement and lymphovascular invasion were also reported to have no prognostic value in other studies.^11^, ^13^, ^18^ Therefore, the prognostic factors in SCENC are different from those of common histological types of cervical cancer.

Brachytherapy acted an important part in the definitive management of common histological types of the uterine cervix.^27^, ^28^ In this study, EBRT plus brachytherapy improved CSS and OS in patients with stage III disease. However, the pelvic control could not be assessed together with the brachytherapy because the endpoint was unavailable in the current database. Whether the addition of brachytherapy could improve pelvic control for SCNEC patients remains controversial. Large database studies with detailed information about disease relapse are required to answer these questions.

Due to its methodology and structure, this study has limitations. First, the information regarding chemotherapy regimens and the sequence with surgery or RT were not available in the SEER database, which may have affected the assessment of the prognostic value of the therapeutic approaches. Second, the SEER database did not provide specific information on clinical or surgical staging hence the conclusions could be affected by staging bias. Third, given that the patients with early stages preferred surgery and patients with advanced stages preferred radiation, the survival outcomes associated with the different modalities would be affected. Lastly, we were unable to exclude the use of palliative RT because there was no information regarding the dose of radiation or the size of the radiation fields in the SEER database.

In conclusion, SCNEC is a rare disease with highly aggressive features and poor survival. This study suggests that surgery would be the optimal local therapeutic approach for early‐stage SCNEC. For patients with advanced stage, combining primary RT with brachytherapy seems to be the better treatment. We hope that our study contributes to the foundation of knowledge regarding this rare and aggressive disease and inspires more prospective studies to help define optimal local management for SCNEC.

## DISCLOSURE

The authors report no conflicts of interest in this work.

## AUTHOR CONTRIBUTIONS

All authors helped to perform the research; Li‐mei Lin manuscript writing and performing procedures; Qin Lin manuscript writing and data analysis; Jun Liu and Ke‐xin Chu contribution to writing the manuscript, drafting conception, and design; Yun‐xia Huang, Zong‐kai Zhang, Tao Li, and Ya‐qing Dai contribution to writing the manuscript and data analysis; Jin‐luan Li contribution to writing the manuscript, drafting conception, and design.

## Data Availability

We registered an account on the official website of the SEER database, signed the agreement and were allowed to access the data. The data in this study are all from the SEER database.
